# Mapping QTLs with additive and epistatic effects for awn length and their effects on kernel-related traits in common wheat

**DOI:** 10.3389/fpls.2024.1417588

**Published:** 2024-08-21

**Authors:** Nina Sun, Wei Liu, Deyang Shi, Chunhua Zhao, Jinlian Ou, Yuanze Song, Zilin Yang, Han Sun, Yongzhen Wu, Ran Qin, Tangyu Yuan, Yanlin Jiao, Linzhi Li, Fa Cui

**Affiliations:** ^1^ Institute of Grain and Oil Crops, Yantai Academy of Agricultural Sciences, Yantai, China; ^2^ Modern Seed Industry and Green Planting & Breeding Research Center, College of Agriculture, Ludong University, Yantai, China

**Keywords:** wheat, QTL, awn length, kernel-related traits, genetic effects

## Abstract

**Introduction:**

Wheat awns are crucial determinants of wheat yield due to their capacity to photosynthesize and exchange gas. Understanding the genetic basis of awn length (AL) is essential for improving wheat yield in molecular breeding programs.

**Methods:**

In this study, quantitative trait loci (QTLs) of AL were analyzed using recombinant inbred line (RIL) mapping population referred to as YY-RILs, which was derived from a cross between Yannong 15 (YN15) and Yannong 1212 (YN1212).

**Results and discussion:**

Seven putative additive QTLs and 30 pairwise epistatic QTLs for AL were identified. Among them, five novel additive QTLs (except *qAl-2A* and *qAl-5A.2*) and 30 novel pairwise epistatic QTLs were identified. *qAl-5A.1* was repeatedly identified in all five environment datasets, which was considered to be one novel stable QTL for AL with minor additive effects. *eqAl-2B.2-2* significantly interacted with eight loci and could be of great importance in regulating awn development. The genes associated with the major stable QTL of *qAl-5A.2* and the minor stable QTL of *qAl-2A* were *B1* and *WFZP-A*, respectively. Awn lengths exhibited significant genetic correlations with kernel weight and kernels per spike, which could affect grain protein content to a lesser extent. This study enhances our understanding of the genetic basis of awn development and identifies novel genes as well as markers for future genetic improvement of wheat yield.

## 1 Introduction

Common wheat (*Triticum aestivum* L.) is a staple food for more than 35% of the world’s population, and its sustainability and adequate supply are vital to food security around the world (Gupta et al., 2008; Liu et al., 2017; Livinus et al., 2021; Nadeem et al., 2023). China is the largest wheat producer and consumer in the world. To meet the demand for feeding the estimated population of approximately 9–10 billion in 2050, wheat production should be increased by approximately 50% over the next two decades (Yang et al., 2021). Therefore, high yield is a pertinent theme for wheat breeding. Wheat spike architecture is one of the key agronomic traits, which considerably influences wheat yield potential (Wang et al., 2022). Among the spike morphological traits, wheat awns have the ability to carry out photosynthesis and gas exchange, which are associated with wheat yield under certain conditions, particularly under abiotic stress conditions (Kjack and Witters, 1974; Motzo and Giunta, 2002; Liller et al., 2017; Masoudi et al., 2019; DeWitt et al., 2020; Niu et al., 2020; Liu et al., 2021). Previous studies have shown that awned cultivars exhibit higher yields than awnless or de-awned wheat cultivars (Vervelde, 1953; Grundbacher, 1963; DeWitt et al., 2020). In addition, because of the biological evolution of traits, wheat awns are crucial in seed dispersal and protection against predators, such as animals and birds (Grundbacher, 1963; Sorensen, 1986; Elbaum et al., 2007). However, during cereal evolution, the awnless or short-awn phenotype was selected due to the capacity of needle-like awns with barbs to prevent manual harvesting (Xiong et al., 1999). The characterization of genes related to the formation of awns can enhance our understanding of the regulatory mechanisms of awn development.

Some progress has been made toward enhancing our understanding of the genetic regulation of awn elongation in rice (*Oryza sativa* L.) as a model crop, and certain genes associated with awn formation have been isolated, such as *An-1* (Luo et al., 2013), *DROOPING LEAF* (*DL*) and *OsETTIN* (*OsETT*) (Toriba and Hirano, 2013), *LONG AND BARBED AWN1* (*LABA1*) (Hua et al., 2015), and *REGULATOR OF AWN ELONGATION 2* (*RAE2*) (Bessho-Uehara et al., 2016). In wheat, three loci, namely, *B1* (*Tipped 1*), *B2* (*Tipped 2*), and *Hd* (*Hooded*), have been identified as dominant suppressors of awn development (Watkins and Ellerton, 1940; Yoshioka et al., 2017; DeWitt et al., 2020; Niu et al., 2020; Wang et al., 2022). The three loci were mapped to chromosome arms 5AL, 4AS, and 6BL (Sourdille et al., 2002; Yoshioka et al., 2017; DeWitt et al., 2020; Wang et al., 2020; Wurschum et al., 2020). The gene associated with the *B1* locus has been cloned as a C2H2 transcription factor with an EAR domain of transcription repression functions (DeWitt et al., 2020; Huang et al., 2020; Wang et al., 2020; Wurschum et al., 2020; Wang et al., 2022). The causal genes associated with *B2* and *Hd* remain unknown due to large linkage disequilibrium intervals in these regions (Wang et al., 2022). The awnless locus of *Anathera* (*Antr*), a novel gene that inhibits awn elongation, has been identified from the wild diploid wheat *Aegilops tauschii* and assigned to the distal region of the chromosome arm 5DS (Nishijima et al., 2018). *WFZP-A* has been isolated from Zang734, an endemic Tibetan wheat variety that exhibits a rare triple spikelet phenotype, and has been demonstrated to simultaneously repress the spikelet formation gene *TaBA1* and activate awn development genes (Du et al., 2021).

In addition to the major genes associated with awn inhibition or elongation, a few quantitative trait loci (QTLs) with minor or moderate effects on awn length (AL) have been documented. Six putative additive QTLs, including *Qa11B-1*, *Qa12D-1*, *Qa12D-2*, *Qal3B-1*, *Qa l5A-1* (*B1*), and *Qal6B-1*, have been identified using two related recombinant inbred line (RIL) mapping populations with Xiaoyan 81 as the common parent (Zhang et al., 2018). Six putative additive QTLs for awn length on chromosomes 1B, 2D, 3B, 4A, and 6A have been identified and reported by Masoudi et al. (2019). A total of 26 single-nucleotide polymorphisms (SNPs) are associated with awn length on chromosomes 1A, 1D, 2A (2), 2B (3), 3A (2), 3B (2), 3D, 4A (2), 4B, 5A (3), 5B (2), 6B (2), 7A (2), and 7D (2) in the 364 wheat accessions (Wang et al., 2020). Three QTLs, *Qawn-1D*, *Qawn-5A* (*B1*), and *Qawn-7B*, have been shown to be associated with awn length based on an F_2_ mapping population with 101 lines derived from a cross between 4045 and Zhiluowumai, the “double-awn” wheat 4045 with super-long lemma awns and long glume awns, awnless Zhiluowumai (Liu et al., 2021). Insertions and deletions (InDels) are associated with awn lengths on chromosomes 3A, 4B, 5D, and 7B with the exception of loci *B1*, *B2*, and *Hd* in the Chinese Wheat Mini-Core Collection; the loci exhibit minimal additive effects (Wang et al., 2022). Epistatic effects are important in the interaction and function of QTLs associated with the regulatory pathways of awn formation; however, a few studies have investigated epistatic effects (Koyama et al., 2001). Two paired epistatic QTLs for awn length have been identified by Masoudi et al. (2019).

Yannong 15 (YN15) is an awnless cultivar, or its top spikelets have extremely short awns, whereas Yannong 1212 (YN1212) has long or moderate awns on all spikelets, particularly on the top spikelets. A RIL mapping population comprising 188 lines derived from a cross between YN15 and YN1212 was developed. The objectives of this study were to 1) identify major and stable QTLs for wheat AL in multiple environments, 2) identify loci with epistatic effects on awn formation, and 3) determine the genetic effect of AL on wheat yield and grain quality traits.

## 2 Materials and methods

### 2.1 Experimental populations and trait evaluation

**Figure 1:**
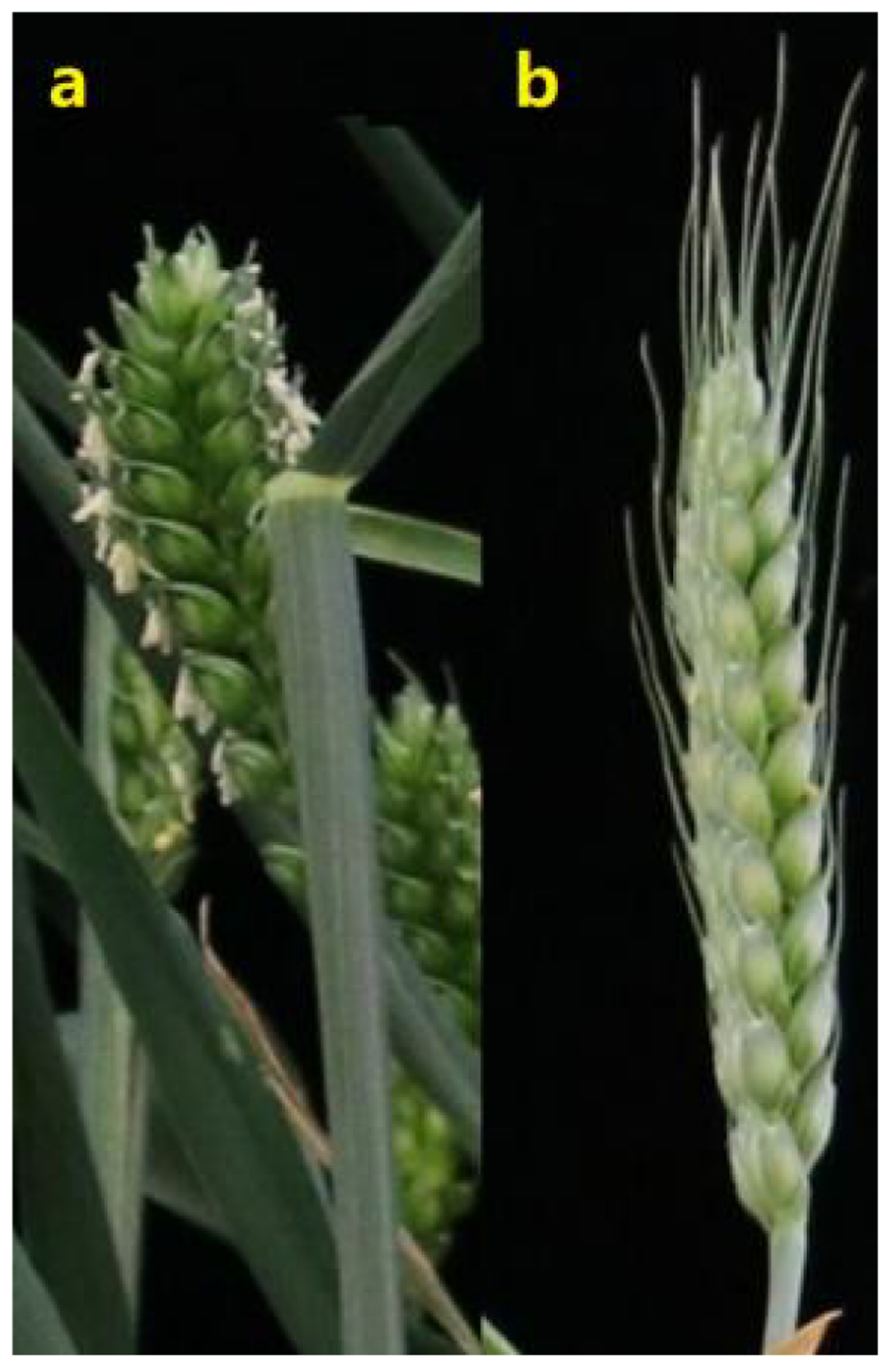
Morphological differences between Yannong 15 **(A)** and Yannong 1212 **(B)** spikes.

The RIL mapping population comprising 188 lines derived from the cross between YN15 and YN1212 (YY-RILs) was developed. Six generations were self-crossed through the single seed descent method, and the F_7:8_ of the 188 YY-RILs was used for phenotype and genotype analyses. The ALs of the two parental lines differed considerably (**Figure 1**). YN15 is an awnless cultivar, or its top spikelets have extremely short awns, whereas YN1212 has long or moderate awns on all spikelets, particularly on the top spikelets.

For the two parents, YN15 was developed by the Yantai Academy of Agricultural Sciences, Yantai, China, in 1982 and has since been promoted and planted for more than 40 years in China due to its excellent processing quality, such as strong wheat flavor and flour whiteness. YN1212 was also developed by the Yantai Academy of Agricultural Sciences in 2018 and is an elite wheat cultivar in China with high yielding potential.

The 188 YY-RILs (F_7:8_) and their parents were grown in four different environments during 2022–2023: Muyu Village, Yantai, Shandong Province, China (E1, 37.33369°N; 121.3568°E; altitude 50 m); Yantai Academy of Agricultural Sciences, Yantai, Shandong Province, China (E2, 37.4859°N; 121.2744°E; altitude 42 m); Boxing County, Binzhou, Shandong Province, China (E3, 37.1547°N; 118.1107°E; altitude 15 m); and Dryland Farming Institute of Hebei Academy of Agricultural and Forestry Sciences, Hengshui, Hebei Province, China (E4, 37.7223°N; 115.7329°E; altitude 18 m). Yantai is a hilly terrain, and the fertility of the soil in different sites varies greatly. The phenotype of the YY-RILs in two different sites in Yantai was evaluated. The soil in E1 is fertile and well-watered, whereas the soil in E2 is poor and dry with limited irrigation. A randomized block design with two replications was used in each of the four environments, and each row was planted with 40 seeds in two plots at a row spacing of 0.25 m and length of 2.0 m. All locally recommended agronomic practices were followed in each of the trials.

To evaluate AL, five representative main stem spikes were sampled from each line of the 188 YY-RILs and their parents. The ALs of the top spikelets in each sampled spike were measured manually at the milk ripening stage and expressed as centimeters. In addition, the thousand kernel weight (TKW) and kernels per spike were evaluated. The grain protein content (GPC) was measured after harvesting by near-infrared reflectance spectroscopy (DA 7200 NIR spectrometer; Perten Instruments, Huddinge, Sweden), according to the method described by Cui et al. (2016). The determination of TKW and GPC was based on all the seeds from the 5–10 representative plants, excluding the shriveled small seeds and the crushed seeds. The GPC was measured three times for the seed samples of each replication in each environment with an FTIR Spectrometer (Thermo Nicolet iS50).

### 2.2 Genotyping and genetic linkage map construction

Genomic DNA for all the 188 YY-RILs, as well as YN15 and YN1212 populations, was extracted from leaf tissues and analyzed using a wheat 55K SNP genotyping array containing 53,063 markers by Compass Biotechnology Company (Beijing, China). The SNPs were deleted if they showed minor allele frequency (defined as frequency <0.3) or had >10% missing data. Markers were binned based on their segregation patterns in the YY-RIL segregating mapping population using the BIN function in IciMapping 4.1 (http://www.isbreeding.net/) according to the method described by Cui et al. (2017). Markers with identical segregation patterns were considered the same informative genetic marker. Only one marker was randomly selected to represent each bin based on the least amount of missing data or when the percentage of missing data was equal. The genetic linkage map was constructed using JoinMap 4.0 according to the method described by Cui et al. (2017). Based on SNP flanking sequences, the SNPs were assigned to IWGSC RefSeq v2.1 according to Cui et al. (2017), with an aim to obtain their physical position.

### 2.3 Data analysis and QTL mapping

Statistical analysis of the phenotypic data for the YY-RIL population was performed using SPSS Statistics 13.0 (SPSS Inc., Chicago, IL, USA). A trait was considered to conform to the normal distribution in the segregating mapping population if both skewness and kurtosis were <1.0 in absolute. The best linear unbiased estimators (BLUEs), the genetic variance (σ^2g^), and the phenotypic variance (σ^2p^) of AL, TKW, kernel number per spike (KNPS), and GPC of the 188 YY-RILs in the four environments were estimated using QGAStation 2.0. The broad heritability of the AL was calculated as *H* ^2^ = σ^2g^/σ^2p^ according to Hanson et al. (1956).

Phenotypic data of ALs in the 188 YY-RILs in each environment and BLUE datasets were used for QTL analysis. Inclusive composite interval mapping of QTLs was performed using QTL IciMapping 4.1 based on stepwise regression of simultaneous consideration of all marker information. The walking speed selected for QTL with additive effects was 1.0 cM, and the *p*-value inclusion threshold was 0.001. The threshold logarithm of odds (LOD) score was manually set at 2.0. Missing phenotypes were replaced by mean values. The walking speed selected for QTLs with epistatic effects was 5.0 cM, and the *p*-value inclusion threshold was 0.001. The threshold LOD score was manually set at 5.0. The genetic effects of the major stable QTL were parsed based on the association analysis between genotypic and phenotypic data in the 188 YY-RILs. Statistical differences were determined using a one-way analysis of variance and Tukey’s test.

## 3 Results

### 3.1 Phenotypic variations of awn length in YY-RILs and the two parents

**Figure 2:**
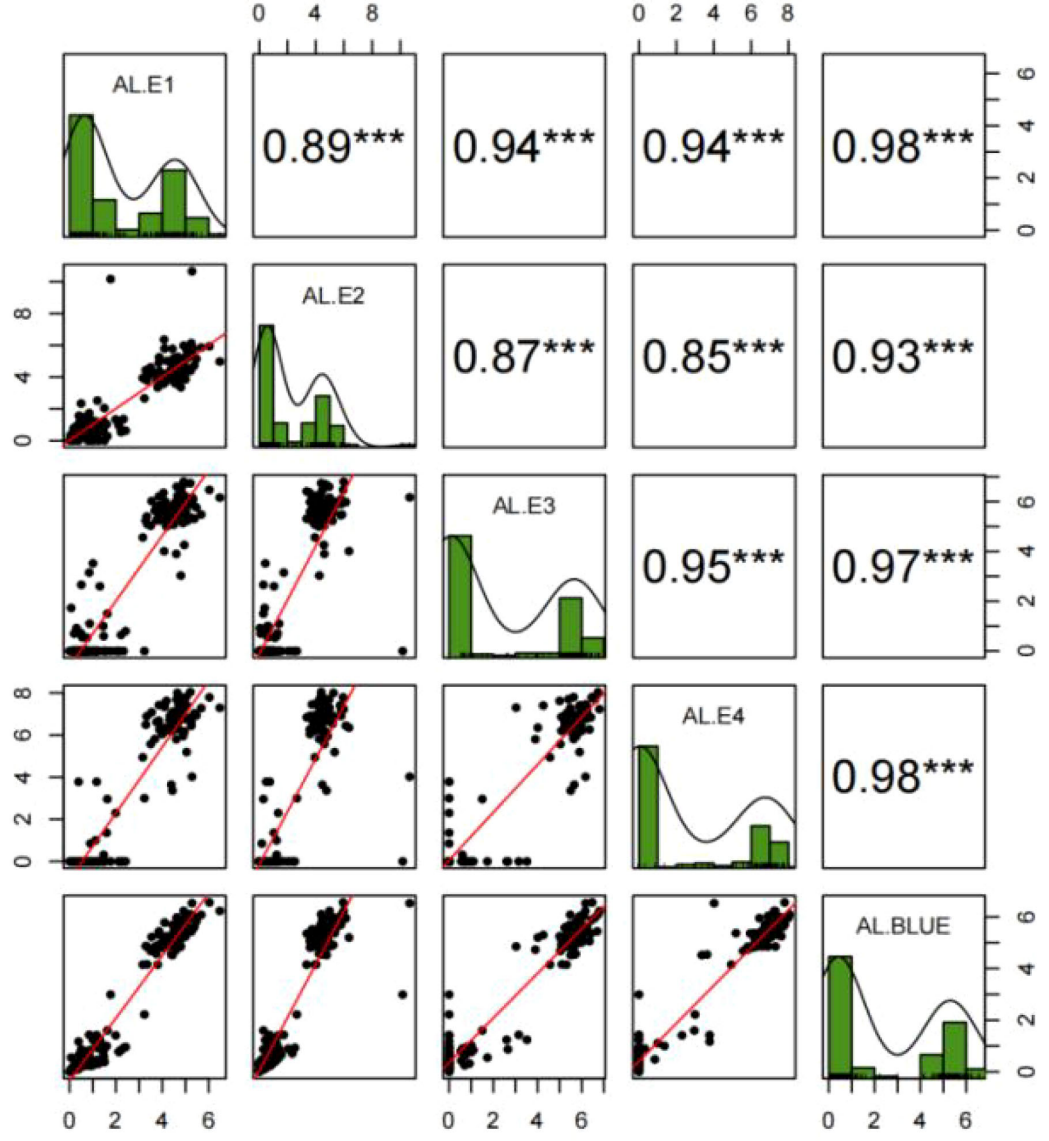
Frequency distributions, correlations, and fitting curves of awn length in the YY-RILs under four environments (E1, E2, E3, and E4) and the BLUE dataset. AL, Awn length. E1, E2, E3, and E4 represent Muyu Village of Yantai, Yantai Academy of Agricultural Sciences, Boxing County of Binzhou City, Dryland Farming Institute of Hebei Academy of Agricultural and Forestry Sciences, respectively, during the 2022–2023 period. BLUE represents the best linear unbiased estimator (BLUE) datasets of awn length based on the values obtained from the four environments. *** indicate significance at *p* < 0.001.

The ALs of YN15 in the four environments (E1, E2, E3, and E4) were in the range of 0–0.68 cm. The ALs of YN1212 in E1, E2, E3, and E4 were approximately 5.24 cm, 4.63 cm, 6.30 cm, and 6.66 cm, respectively. The ALs of the 188 YY-RILs varied substantially across the four environments (**Table 1**). Wheat lines with no awns in the uppermost spikelets were observed in all four environments. The maximum values of ALs in YY-RILs were higher than those in the YN1212 population in the corresponding environments, indicating the existence of alleles with increasing effects on AL in YN15, although the uppermost spikelets of YN15 were awnless (**Table 1**). AL in the 188 YY-RILs under the four environments and the BLUE datasets did not conform to a normal distribution. The results suggest that major genes contribute to the variations in ALs of YN15 and YN1212 populations (**Figure 2**; **Table 1**). The broad heritability for AL ranged from 90.2% to 93.1%, and the correlation coefficients across the four environments ranged from 0.85 to 0.98. The results indicate that genetic factors mainly affect AL (**Figure 1**; **Table 1**).

**Table 1:** Phenotypic variations in wheat awn length in the YY-RIL population across four environments as well as in the BLUE datasets.

Env.	Mean	Variance	Std.	Skewness	Kurtosis	Minimum	Maximum	Range	Heritability (%)
E1	2.28	3.81	1.95	0.40	−1.54	0	6.48	6.48	90.2
E2	2.31	4.77	2.18	0.82	0.14	0	10.66	10.66	92.3
E3	2.38	7.45	2.73	0.40	−1.73	0	6.80	6.8	93.1
E4	2.67	10.55	3.25	0.49	−1.61	0	8.04	8.04	91.2
BLUE	2.41	5.85	2.42	0.41	−1.70	0	6.56	6.56	–

E1, E2, E3, and E4 represent Muyu Village of Yantai, Yantai Academy of Agricultural Sciences, Boxing County of Binzhou City, and Dryland Farming Institute of Hebei Academy of Agricultural and Forestry Sciences during the 2022–2023 period, respectively. The best linear unbiased estimators (BLUEs) of awn lengths in the 188 YY-RILs were estimated based on the values obtained under the four environments. The BLUE datasets were also used for phenotypic analysis. Awn lengths were expressed in centimeters.

### 3.2 YY-RIL-derived genetic linkage map and QTL for awn length

A total of 13,845 SNPs were mapped to the YY-RIL-derived genetic map, and 1,632 SNPs were informative following the exclusion of markers with identical segregating patterns in the YY-RILs. Considering the 1,632 unique informative markers, most markers were mapped to genome A (41.3%), followed by genomes B (30.5%) and D (28.2%). The number of markers on each chromosome ranged from 33 (chromosome 3B) to 136 (chromosome 1A), with a mean of 77.7 loci per chromosome. For the map lengths, the A, B, and D genomes covered 35.2%, 27.1%, and 37.7% of the total map length, respectively. The chromosome sizes ranged from 85.9 cM (chromosome 4B) to 238.0 cM (chromosome 5D), averaging 144.9 cM per chromosome (data not shown).

**Figure 3:**
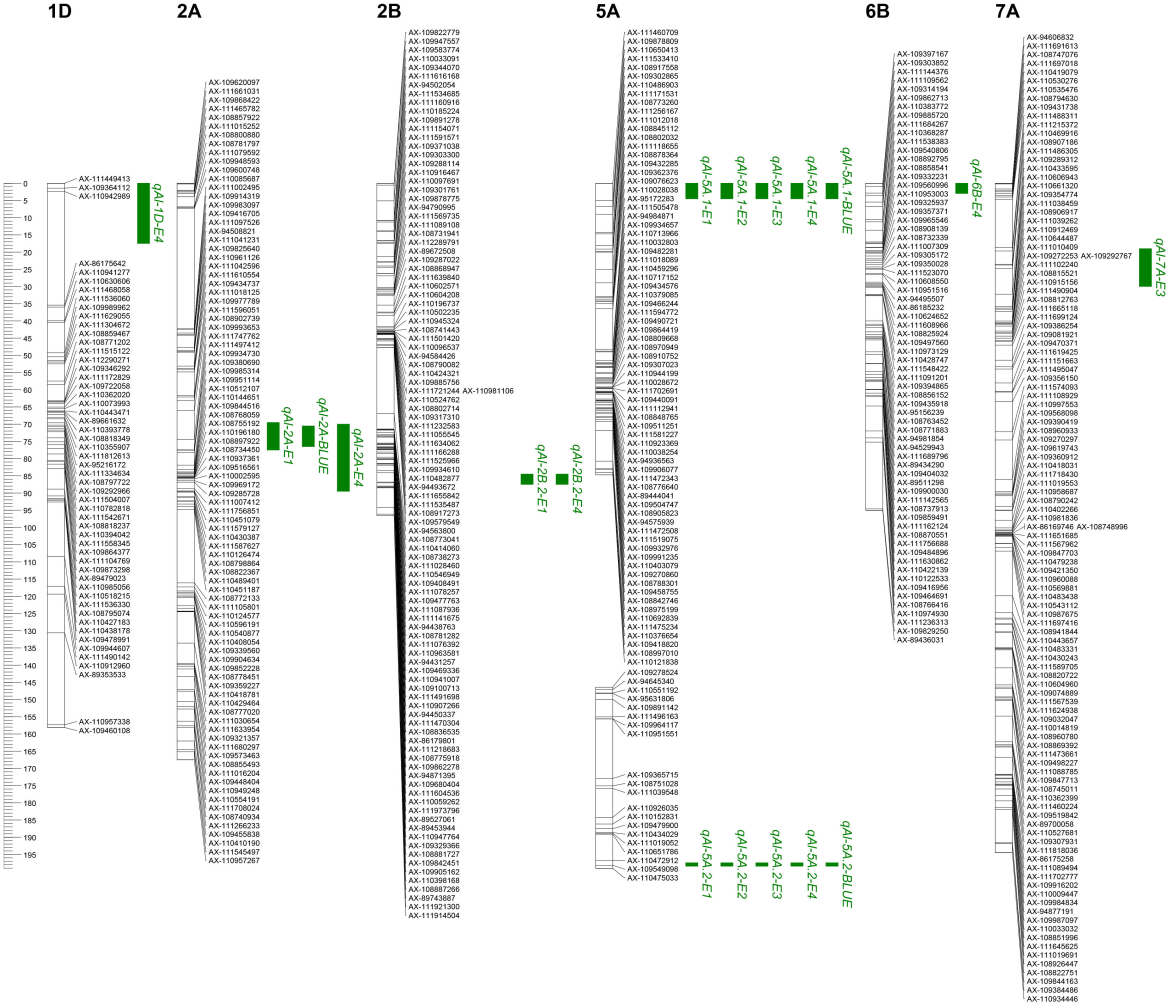
Locations of quantitative trait loci (QTLs) for awn length identified in four environments (E1, E2, E3, and E4) as well as in the best linear unbiased estimator (BLUE) datasets based on the 188 YY-RILs derived from a cross between Yannong 15 and Yannong 112. The short arms of chromosomes are located at the top of the figure. The names of the marker loci and QTLs are listed to the right of the corresponding chromosomes. QTL intervals were based on the logarithm of odds (LOD) scores >2.0, with LOD peak values of more than 2.5 being selected. E1, E2, E3, E4, and BLUE indicate the environments where QTLs were identified. Detailed information is provided in the notes of **Figure 1**.

**Figure 4:**
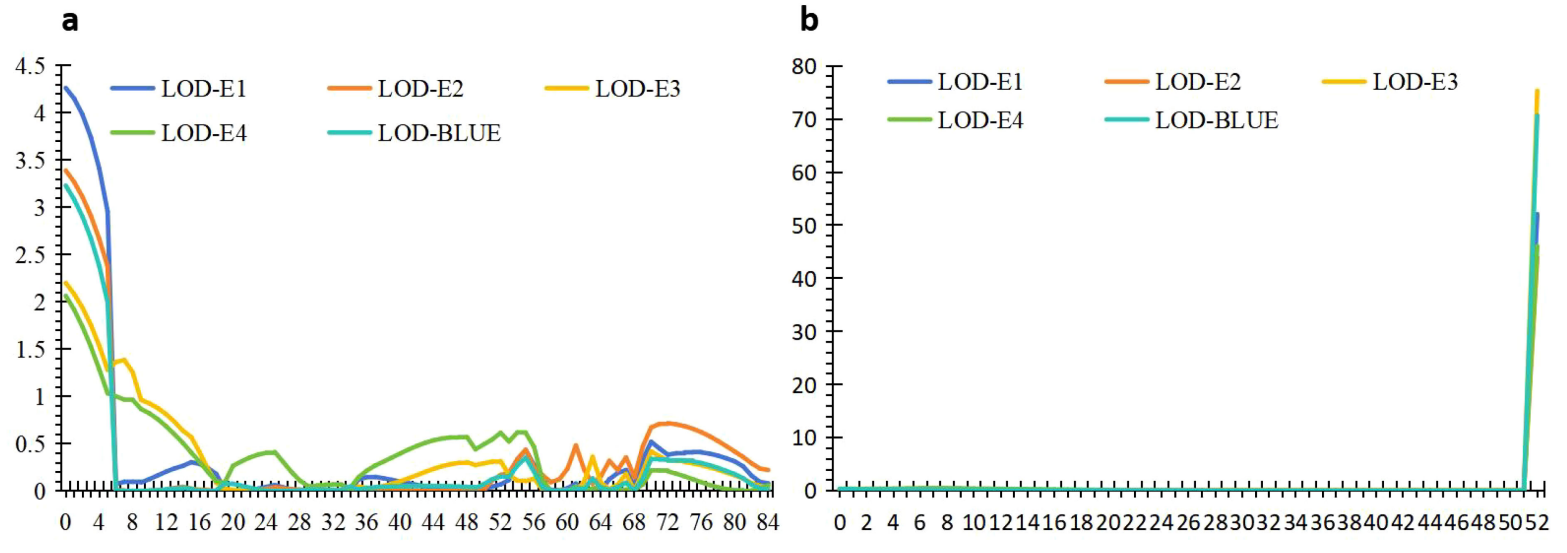
The distribution of logarithm of odds (LOD) values for two stable quantitative trait loci (QTLs), *qAl-5A.1* **(A)** and *qAl-5A.2* **(B)**, in four environments as well as in the BLUE datasets. The abscissa represents the genetic position in the linkage map derived from the YY-RILs, and the ordinate indicates the LOD value corresponding to different environment datasets.

The 1,632 SNPs were used for QTL mapping analysis. Seven putative additive QTLs for AL were identified across the four environments and the BLUE datasets. The QTLs were distributed on chromosomes 1D, 2A, 2B, 5A, 6B, and 7A (**Figure 3**; **Table 2**). The QTL independently explained 0.99%–77.81% of the phenotypic variance of AL in the YY-RILs (**Table 2**). Among them, *qAl-5A.1* and *qAl-5A.2* were repeatedly identified in all five environment datasets, and they accounted for 0.99%–2.83% and 61.98%–77.81% of the variation in AL in the YY-RILs, respectively. LOD peak values of *qAl-5A.1* and *qAl-5A.2* were 2.07–4.26 and 43.94–75.31, respectively (**Figure 4**). Both alleles that decreased AL were from YN15. Therefore, *qAl-5A.2* is a major stable QTL for AL. *qAl-2A* was verified in three of the five environment datasets, and it explained 0.99%–2.21% of the variation in AL in the YY-RILs, with alleles decreasing AL from YN1212. Therefore, *qAl-2A* could be a relatively stable QTL for AL with moderate or minor additive effects. *qAl-2B.2* was identified in two of the five environment datasets, and it explained 1.87%–2.55% of the phenotypic variation, with alleles decreasing AL from YN15. *qAl-1D*, *qAl-6B*, and *qAl-7A* were identified in a unique environment, with low LOD peak values and phenotypic variation being observed. The three QTLs are environment-specific QTLs with minor effects. Notably, four QTLs with additive effects of increasing AL were identified from YN15, the awnless cultivar; however, most were environment-specific QTLs with minor effects.

**Table 2:** Putative additive QTLs for awn length in the YY-RIL population identified by IciMapping 4.0.

QTL	Environ.	Chrom.	Pos. (cM)	Left marker	Right marker	LOD	PVE (%)	ADD.
*qAl-1D*	E4	1D	3.00	*AX-110942989*	*AX-86175642*	2.30	1.76	0.42
*qAl-2A*	E1	2A	75.00	*AX-111747762*	*AX-111497412*	3.24	2.21	0.29
	BLUE	2A	75.00	*AX-111747762*	*AX-111497412*	2.17	0.99	0.26
	E4	2A	89.00	*AX-111007412*	*AX-111756851*	2.38	1.86	0.43
*qAl-2B.2*	E1	2B.2	91.52	*AX-110947764*	*AX-109329366*	2.24	1.87	−0.32
	E4	2B.2	91.52	*AX-110947764*	*AX-109329366*	2.54	2.55	−0.59
*qAl-5A.1*	E1	5A.1	0.00	*AX-111460709*	*AX-109878809*	4.26	2.83	−0.33
	E2	5A.1	0.00	*AX-111460709*	*AX-109878809*	3.39	2.81	−0.38
	E3	5A.1	0.00	*AX-111460709*	*AX-109878809*	2.20	0.99	−0.30
	E4	5A.1	0.00	*AX-111460709*	*AX-109878809*	2.07	1.53	−0.39
	BLUE	5A.1	0.00	*AX-111460709*	*AX-109878809*	3.23	1.52	−0.33
*qAl-5A.2*	E1	5A.2	52.00	*AX-109549098*	*AX-110475033*	52.07	68.52	−1.64
	E2	5A.2	52.00	*AX-109549098*	*AX-110475033*	43.94	61.98	−1.80
	E3	5A.2	52.00	*AX-109549098*	*AX-110475033*	75.31	77.81	−2.66
	E4	5A.2	52.00	*AX-109549098*	*AX-110475033*	46.04	64.62	−2.53
	BLUE	5A.2	52.00	*AX-109549098*	*AX-110475033*	70.65	76.68	−2.37
*qAl-6B*	E4	6B	0.00	*AX-109397167*	*AX-109303852*	2.24	1.65	0.40
*qAl-7A*	E3	7A	23.00	*AX-111215372*	*AX-110469916*	2.24	1.04	0.30

E1, E2, E3, and E4 represent the environments where the corresponding quantitative trait loci (QTLs) were identified; for details, see the notes in **Table 1**. For the additive effects, the positive values indicate an increasing allele from Yannong 15, while the negative values represent the increasing allele corresponding to Yannong 1212.

AL, awn length; BLUE, best linear unbiased estimator.

**Figure 5:**
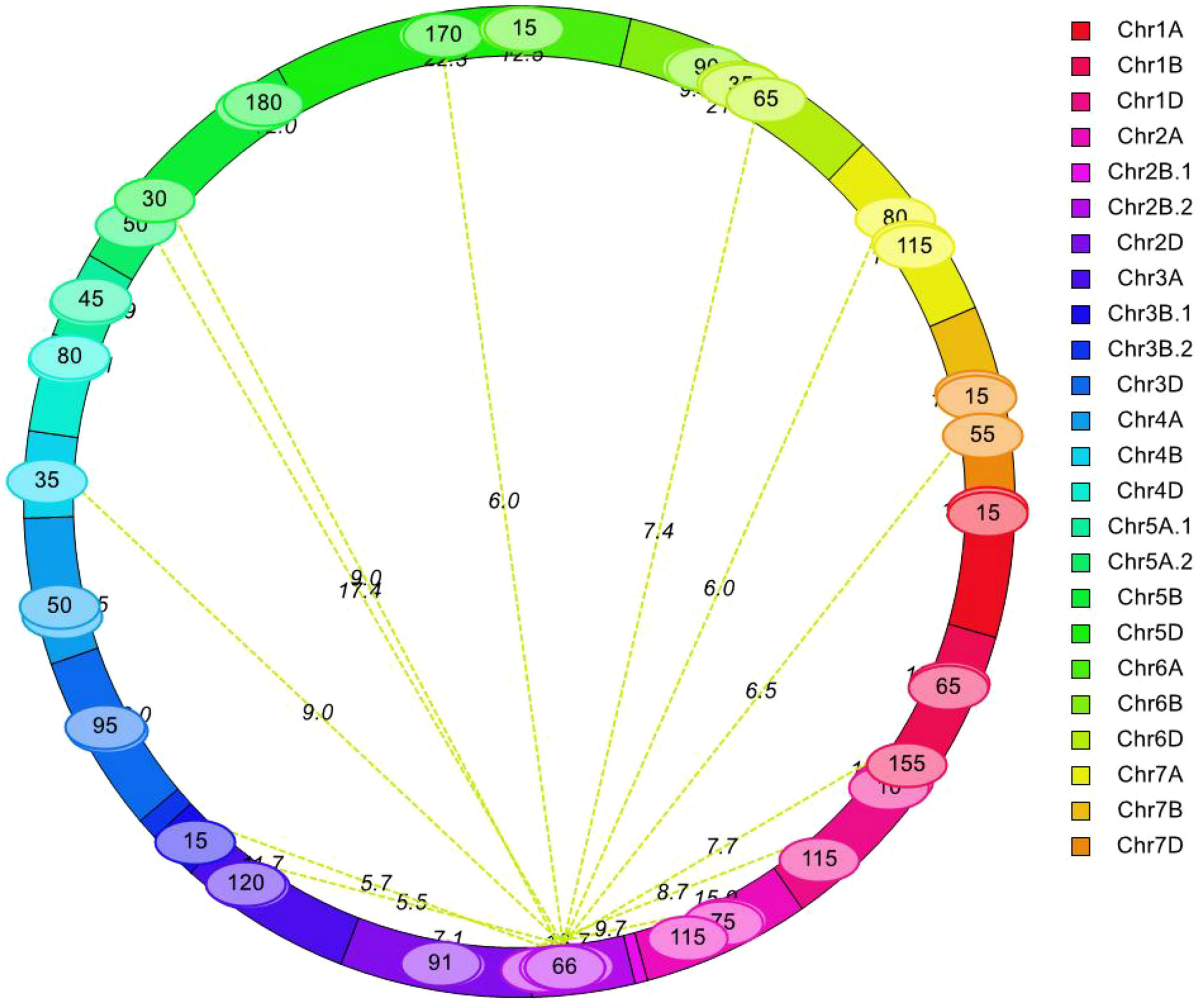
Analysis of epistatic quantitative trait loci (QTLs) for awn length. Values on the line represent logarithm of odds (LOD) values of two interacting QTLs, whereas values in the ellipse represent the genetic position (cM) in the genetic linkage map derived from YY-RILs.

A total of 30 pairwise QTLs with epistatic effects for AL were identified in the YY-RILs based on the BLUE datasets. These epistatic QTLs independently explained 1.83%–3.10% of the variations in AL in the YY-RILs, with LOD peak values ranging from 5.47 to 22.35. The QTLs were distributed on all 21 wheat chromosomes, except 7B (**Figure 5**; **Table 3**). Among the 30 pairwise epistatic QTLs, *qAl-1D*, *qAl-2A*, *qAl-2B.2*, and *qAl-5A.2* showed strong additive effects (**Tables 2**, **3**). *qAl-1D* and *eqAl-1D-1* exhibited significant interactive effects with a LOD value of 15.35 and accounted for 2.23% of the phenotypic variance. *qAl-2A* exhibited significant interactive effects with *eqAl-2A-1* and had moderate additive effects. However, the effects were suppressed due to their interaction, thereby resulting in a considerable reduction in the additive effects, with zero being the actual value. *qAl-2B.2* exhibited significant interactive effects with *eqAl-1D-2*, *eqAl-2A-2*, and *eqAl-3B.1* simultaneously and accounted for 2.04%, 1.83%, and 2.00% of the phenotypic variance, respectively. LOD peak values of *eqAl-1D-2*, *eqAl-2A-2*, and *eqAl-3B.1* were 8.71, 9.68, and 5.66, respectively. *qAl-5A.2* was identified as a major stable QTL for AL, and it exhibited significant interactive effects with *eqAl-2B.2-2*. *qAl-5A.2* accounted for 2.64% of the phenotypic variance and had a LOD value of 17.44 (**Figure 5**; **Table 3**).

**Table 3:** Putative epistatic QTLs for awn length in the YY-RIL population as identified by IciMapping 4.0.

QTL 1	Chrom. 1	Pos. 1	Left Marker 1	Right Marker 1	QTL 2	Chrom. 2	Pos. 2	Left Marker 2	Right Marker 2	LOD	PVE (%)	ADD. 1	ADD. 2	ADD. by ADD.
*eqAl-1A-1*	1A	10.00	*AX-110669146*	*AX-110511743*	*eqAl-1A-2*	1A	15.00	*AX-110669146*	*AX-110511743*	12.75	2.29	0.99	−0.89	1.03
*eqAl-1B-1*	1B	60.00	*AX-111125943*	*AX-108768730*	*eqAl-1B-2*	1B	65.00	*AX-111125943*	*AX-108768730*	18.46	3.01	−1.94	1.91	0.00
*eqAl-1B-3*	1B	155.00	*AX-108890271*	*AX-110444369*	***eqAl-2B.2-3***	2B.2	70.52	*AX-109579549*	*AX-94563800*	7.67	2.26	−0.93	−0.95	−1.09
***qAl-1D***	1D	5.00	*AX-110942989*	*AX-86175642*	*eqAl-1D-1*	1D	10.00	*AX-110942989*	*AX-86175642*	15.35	2.29	0.98	−0.89	1.04
*eqAl-1D-2*	1D	115.00	*AX-109944607*	*AX-111490142*	***qAl-2B.2***	2B.2	90.52	*AX-110947764*	*AX-109329366*	8.71	2.04	0.93	−0.95	1.06
*eqAl-2A-1*	2A	70.00	*AX-109993653*	*AX-111747762*	***qAl-2A***	2A	75.00	*AX-111747762*	*AX-111497412*	15.87	3.10	−1.84	2.01	0.00
*eqAl-2A-2*	2A	115.00	*AX-110451187*	*AX-108772133*	***qAl-2B.2***	2B.2	90.52	*AX-110947764*	*AX-109329366*	9.68	1.83	0.99	−1.02	0.96
*eqAl-2B.2-1*	2B.2	55.52	*AX-109579549*	*AX-94563800*	***eqAl-2B.2-2***	2B.2	65.52	*AX-109579549*	*AX-94563800*	13.74	2.34	0.99	−1.10	0.85
***eqAl-2B.2-2***	2B.2	65.52	*AX-109579549*	*AX-94563800*	*eqAl-3A-2*	3A	120.00	*AX-108973024*	*AX-110402974*	5.47	2.25	−1.02	0.88	1.01
***eqAl-2B.2-2***	2B.2	65.52	*AX-109579549*	*AX-94563800*	***eqAl-5D-1***	5D	165.00	*AX-89753391*	*AX-111323000*	6.00	2.26	−0.88	1.03	1.03
***eqAl-2B.2-2***	2B.2	65.52	*AX-109579549*	*AX-94563800*	*eqAl-7A-1*	7A	80.00	*AX-111108929*	*AX-110997553*	6.02	2.12	−0.85	−1.05	−1.16
***eqAl-2B.2-2***	2B.2	65.52	*AX-109579549*	*AX-94563800*	*eqAl-7D-3*	7D	55.00	*AX-110830564*	*AX-111067707*	6.49	2.26	−0.92	0.98	1.04
***eqAl-2B.2-2***	2B.2	65.52	*AX-109579549*	*AX-94563800*	*eqAl-6D-3*	6D	65.00	*AX-109854414*	*AX-109915708*	7.38	2.46	−0.75	−1.14	−1.16
***eqAl-2B.2-2***	2B.2	65.52	*AX-109579549*	*AX-94563800*	*eqAl-4B*	4B	35.00	*AX-110488730*	*AX-108800362*	9.03	2.39	−0.80	−1.06	−1.18
***eqAl-2B.2-2***	2B.2	65.52	*AX-109579549*	*AX-94563800*	***qAl-5A.2***	5A.2	50.00	*AX-110651786*	*AX-110472912*	17.44	2.64	−0.84	0.90	1.24
***eqAl-2B.2-3***	2B.2	70.52	*AX-109579549*	*AX-94563800*	*eqAl-5B-1*	5B	30.00	*AX-109303068*	*AX-110981573*	8.98	2.26	−0.94	1.00	1.02
***qAl-2B.2***	2B.2	90.52	*AX-110947764*	*AX-109329366*	*eqAl-3B.1*	3B.1	15.00	*AX-109299632*	*AX-111032711*	5.66	2.00	−0.99	−0.97	−0.99
*eqAl-2D-1*	2D	86.14	*AX-109389372*	*AX-108912943*	*eqAl-2D-2*	2D	91.14	*AX-109389372*	*AX-108912943*	7.10	2.18	1.02	−1.00	0.95
*eqAl-3A-1*	3A	115.00	*AX-108973024*	*AX-110402974*	*eqAl-3A-2*	3A	120.00	*AX-108973024*	*AX-110402974*	11.68	2.30	−1.07	0.94	0.96
*eqAl-3D-1*	3D	90.00	*AX-108822945*	*AX-89699893*	*eqAl-3D-2*	3D	95.00	*AX-110687174*	*AX-111571185*	18.04	3.02	1.95	−1.90	0.01
*eqAl-4A-1*	4A	40.00	*AX-110498221*	*AX-108899858*	*eqAl-4A-2*	4A	50.00	*AX-108899858*	*AX-109335789*	18.46	3.03	−1.96	1.88	0.01
*eqAl-4D-1*	4D	75.00	*AX-89453387*	*AX-108786137*	*eqAl-4D-2*	4D	80.00	*AX-108939109*	*AX-109905441*	15.05	2.28	0.89	−0.87	1.09
*eqAl-5A.1-1*	5A.1	40.00	*AX-110028038*	*AX-95172283*	*eqAl-5A-2*	5A.1	45.00	*AX-110028038*	*AX-95172283*	12.90	2.27	1.00	−1.04	0.93
*eqAl-5B-2*	5B	170.00	*AX-95629586*	*AX-108748458*	*eqAl-5B-3*	5B	180.00	*AX-95629586*	*AX-108748458*	11.95	2.26	0.94	−1.00	0.99
*eqAl-5D-1*	5D	165.00	*AX-89753391*	*AX-111323000*	*eqAl-5D-2*	5D	170.00	*AX-89753391*	*AX-111323000*	22.35	3.01	−1.92	1.93	0.00
*eqAl-6A-1*	6A	10.00	*AX-108729212*	*AX-111249450*	*eqAl-6A-2*	6A	15.00	*AX-108729212*	*AX-111249450*	12.47	2.59	1.27	−1.24	0.66
*eqAl-6B-1*	6B	85.00	*AX-111236313*	*AX-109829250*	*eqAl-6B-2*	6B	90.00	*AX-111236313*	*AX-109829250*	9.67	2.25	0.98	−0.98	0.97
*eqAl-6D-1*	6D	30.00	*AX-109823189*	*AX-109346183*	*eqAl-6D-2*	6D	35.00	*AX-109823189*	*AX-109346183*	21.26	3.01	1.73	−1.77	0.17
*eqAl-7A-2*	7A	110.00	*AX-111697416*	*AX-108941844*	*eqAl-7A-3*	7A	115.00	*AX-111697416*	*AX-108941844*	14.37	2.27	0.97	−0.92	1.02
*eqAl-7D-1*	7D	10.00	*AX-109788290*	*AX-110707055*	*eqAl-7D-2*	7D	15.00	*AX-111607406*	*AX-89502768*	13.17	3.01	−1.91	1.93	0.00

“eq” plus the AL and the chromosome name represents the quantitative trait loci (QTLs) only with significant epistatic effects without significant additive effects. The corresponding QTL names shown in **Table 2** that exhibited both significant epistatic and additive effects are still used herein, and are underlined in bold red typeset color. The epistatic QTL with significant interaction effects with more than one locus are underlined in bold black typeset color. For the additive-by-additive interaction effects, the positive values indicate that the two QTLs are the same as those in parent “YN15” (or “YN1212”) taking the positive effect, while the two QTL recombinants take the negative effect. The negative values represent the opposite.

*eqAl-2B.2-2*, *eqAl-2B.2-3*, and *eqAl-5D-1* interacted with more than one locus, and each of them may play a key role in the genetic regulation of awn formation in wheat. *eqAl-2B.2-2* significantly interacted with eight loci (*eqAl-2B.2-1*, *eqAl-3A-2*, *eqAl-5D-1*, *eqAl-7A-1*, *eqAl-7D-3*, *eqAl-6D-3*, *eqAl-4B*, and *qAl-5A.2*), and their LOD peak values ranged from 5.47 to 17.44. The interactive effects of *eqAl-2B.2-2* explained 18.72% of the variations in AL in the 188 YY-RILs. *eqAl-2B.2-3* exhibited significant interactive effects with *eqAl-1B-3* and *eqAl-5B-1*, which had LOD peak values of 7.68 and 8.98, respectively. *eqAl-1B-3* and *eqAl-5B-1* explained 2.26% of the phenotypic variance in the YY-RILs. *eqAl-5D-1* exhibited significant interactive effects with *eqAl-2B.2-2* and *eqAl-5D-2*, which had LOD peak values of 6.00 and 22.35, respectively. *eqAl-2B.2-2* and *eqAl-5D-2* accounted for 2.26% and 3.01% of the variations in AL in the 188 YY-RILs, respectively (**Figure 5**; **Table 3**).

Regarding the additive-by-additive interactive effects, only five out of 30 paired epistatic QTLs had negative values, indicating that most of the parental genotypes (83.3%) or non-recombinants increased AL for the corresponding pairs of interacting loci (**Figure 5**; **Table 3**).

### 3.3 Genetic relationships among awn length, yield, and grain quality

The 188 YY-RILs can be classified into two categories: awnless (AL, 0–0.93 cm) and awned (AL, 4.15–6.56 cm). There were 100 awnless and 75 awned wheat lines in the 188 YY-RILs across the four environments based on the BLUE datasets. ALs of the remaining 13 YY-RILs ranged from 0.96 to 2.99 cm, which could be regarded as recombinants. The top 70 lines with the shortest AL (0–0.38 cm) and the longest AL (4.57–5.56 cm) were used as samples to characterize the effects of AL on wheat yield and grain quality traits. The results showed that AL significantly affected TKW and KNPS. The TKW and KNPS of awned wheat lines increased by 7.34% and 2.74%, respectively, when compared to those of the awnless wheat lines (**Table 4**). Moreover, GPC in awned wheat lines increased by 0.21%, although the increase was not significant (**Table 4**).

**Table 4:** Effects of awn length on wheat yield and grain quality traits in the YY-RIL population.

Type	No. lines	AL (cm)	KNPS	SpNPS	TKW (g)	GPC (%)
Awnless	70	0.24 ± 0.10a	63.10 ± 4.66a	19.33 ± 0.92a	41.89 ± 2.85a	14.29 ± 0.22a
Awned	70	5.38 ± 0.45b	61.37 ± 4.27b	19.17 ± 0.94a	44.95 ± 2.70b	14.32 ± 0.23a
Increased rates (%)	–	–	–2.74	–0.81	7.34	0.21

AL, awn length; KNPS, kernel number per spike; SpNPS, spikelet number per spike; TKW, thousand kernel weight; GPC, grain protein content. The increased rates were calculated as: Increased rates = 100 × (value of the awned wheat line–value of the awnless wheat line)/value of the awnless wheat line. Only the BLUE environment datasets were used for this analysis. Awnless, the YY-RILs with awn lengths of 0–0.38 cm; Awned, the YY-RILs with awn lengths of 4.57–5.56 cm.

If the two sets of data both contain lowercase letter a, it indicates no significant difference between them. Otherwise, if the two sets of data include a and b, it indicates significant difference between the two sets of data at the level of of P < 0.05.

**Figure 6:**
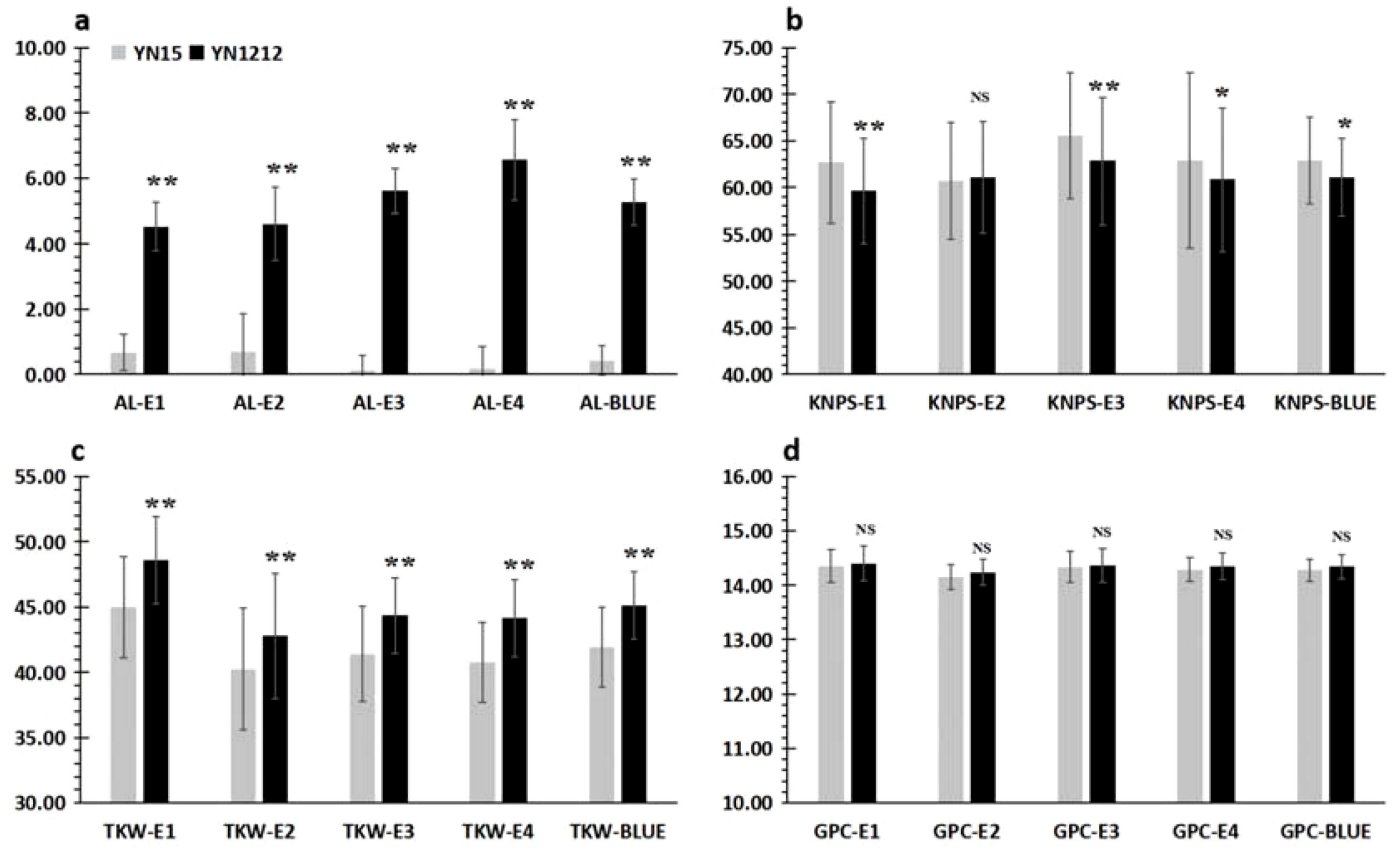
Genetic effects of *qAl-5A.2* on yield and quality-related traits of wheat grown in four environments (E1, E2, E3, and E4) as well as in the BLUE datasets of the YY-RILs. AL, awn length; KNPS, kernel number per spike; TKW, thousand kernel weight; GPC, grain protein content. * and ** represent significant differences at *p* < 0.05 and *p* < 0.01, respectively; NS, not significant. The bar chart in gray and black represent the phenotypic values of the RILs with genotypes identical to Yannong 15 and Yannong 1212, respectively. The detailed phenotypic value information for AL, KNPS, TKW and GPC are shown in **(A–D)**, respectively.

*qAl-5A.2* was the only major stable QTL detected for AL in the YY-RILs, accounting for 61.98%–77.81% of AL variations (**Figure 4**; **Table 2**). The 188 YY-RILs were divided into two groups based on the genotypes of the two closely linked markers with alleles from YN15 and YN1212. The YY-RILs with alleles from YN15 had shorter awns (0.12–0.67 cm) than those from YN1212 (4.52–6.56 cm), which showed the awn inhibition effect of *qAl-5A.2* (**Figure 6**). The alleles from YN15 at *qAl-5A.2* significantly increased KNPS in four out of the five environment datasets but significantly decreased TKW in all of the five environment datasets (**Figure 6**). *qAl-5A.2* had no significant effect on GPC; however, GPC in the YY-RILs with alleles from YN15 was consistently lower than that from YN1212 in all of the five datasets.

## 4 Discussion

### 4.1 Genetic relationships among awn length, yield, and grain quality

Wheat awns have the ability to conduct photosynthesis and gas exchange and can therefore improve wheat yield (Grundbacher, 1963; Kjack and Witters, 1974; Motzo and Giunta, 2002; Li et al., 2006; Tambussi et al., 2007; Ali et al., 2010; Maydup et al., 2014). The location of awns on wheat heads facilitates the movement of carbohydrates into kernels due to their relatively short translocation distance (Evans et al., 1972; Li et al., 2006; Ali et al., 2010). In addition, awns play key roles under biotic and abiotic stresses, as they enhance photosynthesis when leaf senescence occurs early or leaves are damaged (Evans et al., 1972; Tambussi et al., 2007).

We performed the genetic effect analysis of the awn length on yield and quality traits (**Figure 6**; **Table 4**). The results showed that the awned wheat lines had significantly higher TKW than that of the awnless wheat lines, which is consistent with the findings of previous studies (Wang et al., 2022). This finding further confirmed that wheat awns have the ability to conduct photosynthesis and thus can improve wheat yield. The genetic effects of the awn length on KNPS were inconsistent as shown in **Table 4** and **Figure 6**, indicating a complex genetic regulatory network between them. Moreover, GPC in awned wheat lines was increased consistently, although the increase was not significant. To date, no study has investigated the relationship between wheat awn and grain quality. The results above demonstrated that wheat AL exerted strong genetic effects on grain weight. However, wheat awn exerted moderate effects on kernels per spike and minor effects on GPC.

### 4.2 Role of epistatic effects on awn length

Epistatic effects are crucial for complex trait development (Ma et al., 2005; Rebetzke et al., 2007; Xu and Crouch, 2008). In recent years, more efforts have been directed toward testing the epistatic effects of QTLs. However, a few epistatic QTLs for wheat AL have been reported. Two pairs of epistatic QTLs with minor effects on chromosomes 3A, 7B, 3B, and 5D for wheat AL have been identified by Masoudi et al. (2019); however, none of these loci showed substantial additive effects. The finding suggests that loci without substantial additive effects could also affect wheat awn formation, as they have moderate or minor effects. In the present study, up to 30 pairwise QTLs with epistatic effects for wheat AL were identified. These epistatic QTLs independently accounted for a small proportion of the variations with minor or moderate effects (**Figure 5**; **Table 3**). The results revealed the important role played by epistatic effects in awn formation. Notably, four out of the seven key QTLs for AL, such as *qAl-1D*, *qAl-2A qAl-2B.2*, and *qAl-5A.2*, exhibited significant interactive epistatic effects with high LOD values. For example, a major stable QTL, *qAl-5A.2*, exhibited significant interactions with *eqAl-2B.2-2*, which had a LOD value of 17.44 and accounted for 2.64% of the phenotypic variance. The results indicate that other loci with interactive effects should be taken into consideration when evaluating the additive effects of the additive QTL. Three QTLs, including *eqAl-2B.2-2*, *eqAl-2B.2-3*, and *eqAl-5D-1*, interacted with more than one locus and could influence the genetic regulation of awn formation in wheat. Notably, *eqAl-2B.2-2* significantly interacted with eight loci. The three loci, particularly *eqAl-2B.2-2*, requires further study to determine their application potential in the future genetic improvement of wheat awn.

### 4.3 Comparison of the present and previous studies

To date, several putative additive QTLs for AL, which are distributed on chromosomes 1A, 1B, 1D, 2A, 2B, 2D, 3A, 3B, 4A, 4B, 5A, 5B, 6A, 6B, 7A, 7B, and 7D, have been reported (Zhang et al., 2018; Masoudi et al., 2019; Wang et al., 2020; Liu et al., 2021; Wang et al., 2022). Most QTLs for AL on chromosomes 4A, 5A, and 6B corresponded to positions *B2*, *B1*, and *Hd*, which are the three key genes associated with awn formation.

In this study, AL in the 188 YY-RILs did not conform to a normal distribution, indicating that major genes may contribute to the variations in ALs of the YY-RILs mapping populations rather than the numerous genes with minor or moderate effects (**Figure 2**). A total of seven putative additive QTLs distributed on chromosomes 1D, 2A, 2B, 5A (2), 6B, and 7A were identified for AL in this study. Among them, *qAl-5A.2* was the only major stable QTL that explained 61.98%–77.81% of the variations in AL, with LOD peak values of 43.94–75.31; this is consistent with the phenotypic frequency distribution of the AL in the 188 YY-RILs. Regarding the physical position of *qAl-5A.2*, its LOD peak position was at chr5A:700.0–700.5 Mb (IWGSC RefSeq V2.1); *B1* was mapped to chr5A:700.8 Mb (*TraesCS5A02G542800*); therefore, *qAl-5A.2* should be the result of allelic variation in *B1*. Another stable QTL of *qAl-5A.1* was identified in all five environment datasets, and its genetic position suggested that it was located on chromosome arm 5AS. However, the QTL was located on 5AL based on the physical position, which was approximately 7.0 Mb away from *qAl-5A.2*. Therefore, variation in the chromosome structure may occur in this chromosome region. Three QTLs of *qAL.5A.1*, *qAL.5A.2*, and *qAL.5A.3* for AL were identified by Wang et al. (2020) on chromosome 5A; *qAL.5A.3* was proved to be the gene of *B1*; *qAL.5A.1* and *qAL.5A.2* were approximately 635.5 Mb and 152.5 Mb away from *qAL.5A.3*, respectively (Wang et al., 2020). No other QTL for AL on chromosome 5A has been seen so far. *QAl-2A* QTL was mapped to the physical interval of chr2A:58.6–181.4 Mb (IWGSC RefSeq V2.1), which overlapped with *trs1/WFZP-A* (*TraesCS2A02G116900*; IWGSC RefSeq V2.1, chr2A:71.5 Mb) (Du et al., 2021); therefore, *qAl-2A* could be associated with an allelic variation from the known gene of *trs1/WFZP-A*. The LOD peak position of *qAl-1D* was at chr1D:9.0 Mb (IWGSC RefSeq V2.1), which was approximately 6.0 Mb away from *qAL.1D* as reported by Wang et al. (2020); *Kukri_c94613_316* and *Excalibur_c8161_1443* were proved to be significantly associated with AL by Liu et al. (2021), which were located at chr1D:496.8 Mb and chr1D:488.7 Mb (IWGSC RefSeq V2.1), respectively; no other QTL for AL was reported on chromosome 1D. *qAl-2B.2* was mapped at chr2B:691.0 Mb (IWGSC RefSeq V2.1), which were approximately 633.2 Mb, 269.7 Mb, and 105.1 Mb away from *qAL.2B.1*, *An-1*, and *qAL.2B.3* as reported by Wang et al. (2020), respectively; no other QTL for AL on chromosome 2B has been documented to date. *qAl-6B* was mapped at chr6B:21.2 Mb (IWGSC RefSeq V2.1), which was 693.3 Mb and 708.5 Mb away from *qAL.6B.1_B2* and *qAL.6B.2* as reported by Wang et al. (2020), respectively; *Qal6B-1* was mapped at chr6B:146.6–152.0 Mb by Zhang et al. (2018); no other QTL for AL on chromosome 6B has been seen by now. *qAl-7A* was mapped to chr7A:35.4–38.5 Mb (IWGSC RefSeq V2.1), and *Lks2* was mapped at chr7A:595.6 Mb (Wang et al., 2020); no other QTL for AL on chromosome 7A has been seen so far. To sum up, *qAl-1D*, *qAl-2B.2*, *qAl-5A.1*, *qAl-6B*, and *qAl-7A* should be novel additive QTLs for AL that were first documented in this study.

Regarding the QTLs with epistatic effects for AL, only two pairwise QTLs have been documented so far, which were *QAL-3A*/*QAL-7B* and *QAL-3B*/*QAL-5D* (Masoudi et al., 2019). Judging based on the DNA sequence of the flanking markers, *QAL-3A*, *QAL-3B*, *QAL-5D*, and *QAL-7B* were at chr3A:9.6 Mb, chr3B:844.7 Mb, chr5D:403.8 Mb, and chr7B:722.9 Mb, respectively (IWGSC RefSeq V2.1). In this study, *eqAl-3A-1*, *eqAl-3A-2*, *eqAl-3B.1*, and *eqAl-5D* were mapped to chr3A:710.0–718.5 Mb, chr3A:710.0–718.5 Mb, chr3B:21.7–63.6 Mb, and chr5D:502.7–521.8 Mb, respectively. Therefore, all 30 pairwise epistatic QTLs should be first documented in this study.

In summary, seven putative additive QTLs and 30 pairwise QTLs with epistatic effects for AL were identified. Five of the additive QTLs and 30 pairwise QTLs with epistatic effects are reported for the first time in this study. The gene associated with *qAl-5A.2*, which was a major stable QTL, was identified as *B1*. The gene associated with *qAl-2A* was identified as *WFZP-A*, while *eqAl-2B.2-2* significantly interacted with eight loci and could considerably influence the regulation of wheat awn development. AL was significantly and genetically associated with TKW and KNPS, and it affected GPC to a lesser extent. The findings of this study will enhance our understanding of the genetic basis of wheat awn development and present novel genes as well as molecular markers for the genetic improvement of wheat yield.

## Data Availability

The original contributions presented in the study are included in the article/supplementary material. Further inquiries can be directed to the corresponding authors.
